# Gene Network Analysis for Osteoporosis, Sarcopenia, Diabetes, and Obesity in Human Mesenchymal Stromal Cells

**DOI:** 10.3390/genes13030459

**Published:** 2022-03-03

**Authors:** Yilan Jin, Dowan Kim, Yong Jun Choi, Insun Song, Yoon-Sok Chung

**Affiliations:** 1 Department of Endocrinology and Metabolism, Ajou University School of Medicine, Suwon 16499, Korea; jinyilan520@hanmail.net (Y.J.); colsmile@hanmail.net (Y.J.C.); song1009@gmail.com (I.S.); 2 Ajou Institute on Aging, Ajou University Medical Center, Suwon 16499, Korea; 3 Ajou Translational OMICS Center, Ajou University School of Medicine, Suwon 16499, Korea; kdowan@ajou.ac.kr; 4 Department of Medical Sciences, Ajou University Graduate School of Medicine, Suwon 16499, Korea

**Keywords:** osteoporosis, gene network analysis, mesenchymal stromal cells

## Abstract

The systemic gene interactions that occur during osteoporosis and their underlying mechanisms remain to be determined. To this end, mesenchymal stromal cells (MSCs) were analyzed from bone marrow samples collected from healthy individuals (*n* = 5) and patients with osteoporosis (*n* = 5). A total of 120 osteoporosis-related genes were identified using RNA-sequencing (RNA-seq) and Ingenuity Pathway Analysis (IPA) software. In order to analyze these genes, we constructed a heatmap of one-way hierarchical clustering and grouped the gene expression patterns of the samples. The MSCs from one control participant showed a similar expression pattern to that observed in the MSCs of three patients with osteoporosis, suggesting that the differentiating genes might be important genetic determinants of osteoporosis. Then, we selected the top 38 genes based on fold change and expression, excluding osteoporosis-related genes from the control participant. We identified a network among the top 38 genes related to osteoblast and osteoclast differentiation, bone remodeling, osteoporosis, and sarcopenia using the Molecule Activity Predictor program. Among them, 25 genes were essential systemic genes involved in osteoporosis. Furthermore, we identified 24 genes also associated with diabetes and obesity, among which 10 genes were involved in a network related to bone and energy metabolism. The study findings may have implications for the treatment and prevention of osteoporosis.

## 1. Introduction

Osteoporosis is a common, but severe, systemic skeletal disease caused by low bone mass, which results in bone fragility and results in increased risk of hip, spine, wrist, and other bone fractures [1,2]. Risk factors such as aging, low body weight, smoking, alcoholism, and a history of fracture contribute to osteoporosis development [3]. Osteoporosis is characterized by reduced bone mineral density (BMD), deteriorated bone tissue microarchitecture, and a low amount and lack of variety in altered bone proteins [4]. However, systemic gene interactions and molecular mechanisms underlying osteoporosis remain unelucidated.

Obesity is a global health concern. In 2016, over 650 million adults worldwide were classified as obese, with high-energy food intake and physical inactivity considered as the main causes of obesity and overweight [5]. Numerous studies have reported that certain genes affect the progression of obesity, including peroxisome proliferator-activated receptor γ (*PPARG*), CCAAT enhancer binding protein α (*C/EBPα*), and interleukin 6 (*IL6*) [6,7,8]. Moreover, adiponectin, a protein secreted by adipocytes that is involved in regulating glucose levels and lipid metabolism, can be regulated by osteoblasts [9]. Mesenchymal stromal cells (MSCs) can be isolated from various tissues and differentiated into multiple mesenchymal families, such as osteogenic, chondrogenic, adipogenic, and neurogenic cells [10,11]. In addition, the transcriptional profile of osteoporotic stem cells contains numerous genes that predispose individuals to osteoporosis [12]. Osteogenesis and adipogenesis are controlled by the Wnt signaling pathway [13], and nuclear receptor subfamily 4A (*NR4A*) is commonly associated with osteoporosis and obesity [14].

Osteoporosis is a common complication of diabetes mellitus, and patients with diabetes usually have skeletal disorders [15,16,17]. Diabetic osteopathy, an important comorbidity of both type-1 and type-2 diabetes mellitus, increases the risk of bone fracture [18]. Mutation in the gene encoding lipoprotein receptor-related protein 6 (*LRP6*), a co-receptor in the Wnt signaling pathway, is associated with osteoporosis, diabetes mellitus, and coronary artery disease [19].

Although previous studies have investigated the individual mechanisms responsible for the development of osteoporosis, obesity, and diabetes, the common molecular mechanisms underlying the development of these conditions have not yet been determined. In addition, systematic genetic analyses of endocrine diseases need to be performed. Therefore, the aim of the current study was to identify the genetic network related to osteoporosis using next generation sequencing to analyze MSCs isolated from skeletal bone marrow samples collected from healthy individuals with normal bone mineral density (BMD) and patients with osteoporosis. Subsequently, we aimed to discover the network comprised of differentially expressed genes (DEGs) related to osteoporosis, diabetes, and obesity using Ingenuity Pathway Analysis, thus elucidating the relationship of these genes with osteogenesis and adipogenesis.

## 2. Materials and Methods

### 2.1. Study Subjects

Ten subjects aged 40 to 85 years, regardless of gender and with and without osteoporosis, were enrolled in the present study. MSCs were obtained from samples of femur bone marrow collected from participants during total hip or knee arthroplasty due to osteoarthritis, hip fracture, or traffic-related accident. Subjects who presented with other metabolic disorders and secondary causes of osteoporosis were excluded. BMD was measured using dual-energy X-ray absorptiometry (iDXA, GE Lunar, Madison, WI, USA). Subjects with osteoporosis had vertebral fractures or hip fractures and low BMD (T-scores of the lumbar spine, femoral neck, and total hip were ≤−2.5). The five subjects in the control group had no prior fractures and normal BMD (T-scores of the lumbar spine, femoral neck, total hip, and whole BMD were ≥−1.0). The obesity criterion was BMI ≥ 25 kg/m^2^ based on the guidelines of the Korean (Asian) Society for the Study of Obesity [20]. Subjects with osteoporosis and type-2 diabetes mellitus were also enrolled in the study if the onset of diabetes was >30 years of age and supported by medical history/records.

### 2.2. Human MSC Isolation and Culture

Human MSCs were isolated by negative immunoselection (RosetteSep Isolation Kit, STEMCELL Technologies Inc., Vancouver, BC, Canada) according to manufacturer’s instructions. Bone marrow samples were incubated at room temperature (20–22 °C) for 25 min with a depletion cocktail of tetrameric antibodies. The samples were then diluted in 1× phosphate-buffered saline and isolated by density-gradient centrifugation (CR3-22, Jouan, Saint Herblain, France). Isolated human MSCs were seeded into vented 25 cm^2^ tissue culture flasks (Thermo Fisher Scientific, Waltham, MA, USA) containing Complete MesenCult^®^ Medium (human) and MesenCult^®^ MSC Basal Medium (human) mixed with Mesenchymal Stem Cell Stimulatory Supplements (human) (STEMCELL Technologies Inc.) and streptomycin (Welgene, Gyeongsan, Korea). The flasks were incubated in a humidified atmosphere containing 5% CO₂ at 37 °C, and half of the medium was replaced with fresh medium every 3 days. When cells reached 90% confluence, the adherent cells were subcultured in a 100 mm^2^ culture dish (first passage) and re-seeded into three 100 mm^2^ culture dishes (second passage). Once cells reached confluence at the end of the second or third passage, they were used immediately or cryo-preserved [21].

### 2.3. RNA Isolation

Total RNA content was extracted from second or third passaged human MSCs using TRIzol reagent (Invitrogen, Carlsbad, CA, USA) following the manufacturer’s instructions and quantified using a Nanadrop1000 spectrophotometer (Thermo Fisher Scientific, Wilmington, DE, USA). The resulting complete RNA was used for RNA-sequencing.

### 2.4. RNA Library Preparation and Sequencing

To construct cDNA libraries, 1 µg of total RNA was used with the TruSeq strand-specific mRNA library kit (Illumina Inc., San Diego, CA, USA) to achieve polyA-selected RNA extraction, RNA fragmentation, random hexamer primed reverse transcription, and 100 nt paired-end sequencing using the HiSeq 4000 sequencing system (Illumina Inc.). The cDNA libraries were quantified by using quantitative polymerase chain reaction (qPCR) according to the qPCR Quantification Protocol Guide, and library quality was assessed using the 2100 Bioanalyzer (Agilent Technologies, Palo Alto, CA, USA).

To estimate expression levels, RNA-seq reads were mapped to the human genome using TopHat version 1.3.3 software (accessed on 2 August 2018) [22]. The human reference genome sequence (hg19) and annotation data were downloaded from the University of California Santa Cruz Genome Browser website (http://genome.uscs.edu, accessed on 2 August 2018). The transcript counts were calculated at the gene level, the relative transcript abundance was reported in terms of fragments per kilobase of exon per million (FPKM), and fragments were mapped using Cufflinks version 1.2.1 software [23].

### 2.5. Analysis of Gene Expression

Relative gene abundance was calculated in Read Count using StringTie. Statistical analysis was performed to identify DEGs using the estimates of abundance for each gene in the sample. Sample genes that had Read Count values of one more than zero were excluded. The statistical significance of the DEG set was assessed using the edgeR package in Bioconductor and fold change with a null hypothesis showed that no difference existed between groups.

#### 2.5.1. Hierarchical Clustering

Hierarchical clustering analysis was performed using complete linkage and Euclidean distance as a measure of similarity to show the expression patterns of DEGs that satisfied the conditions of |fold change| ≥ 2 and raw *p* < 0.05.

#### 2.5.2. Network Analysis

To construct pathways between genes of interest and biofunctions, we used the Path Explorer tool in Ingenuity Pathway Analysis (IPA) software (QIAGEN Inc., Hilden, Germany) (https://www.qiagenbioinformatics.com/products/ingenuity-pathway-analysis, accessed on 2 June 2021). Pathways were connected by adding intermediate molecules from the QIAGEN Knowledge Base. The Molecule Activity Predictor tool was used to identify downstream effects. Red and pink nodes indicated increased expression of DEGs, while green nodes indicated reduced expression. Orange nodes indicated predicted gene activation and inhibition, whereas blue nodes indicated biofunctions and diseases. The degree of expression or prediction was indicated by node color intensity. Solid lines indicated direct interaction, whereas dashed lines suggested possible gene interaction.

### 2.6. Statistical Analysis

We performed statistical analyses with R software, version 4.0.1 (R Foundation for Statistical Computing, Vienna, Austria). Participant data of clinical characteristics were converted to standardized score (Z-score) and then calculated by the univariate linear regression model with age. To translate the data converted to Z-score to the original scale, an age-adjusted value was multiplied by the sample standard deviation of raw data, and the average value of raw data was added.

## 3. Results

### 3.1. Clinical Characteristics of Study Participants

We compared MSCs extracted from the bone marrow of five patients with osteoporosis (osteoporosis group) with those extracted from the bone marrow of five healthy individuals with normal BMD (control group) (Table 1 (a). The mean weights and BMI values of the osteoporosis group were also lower than those of the control group. Additionally, most participants in the osteoporosis group were diagnosed with type 2 diabetes. There was a significant difference between control and osteoporosis patients with lumbar spine, femur neck, total hip, and whole-body BMD values in age-adjusted analysis data (Table 1 (b)). Other variables including sex, diabetes, obesity, and menopause did not show any significance.

### 3.2. Comparison of Gene Expression Patterns between Control and Osteoporosis Groups

**Figure 1:**
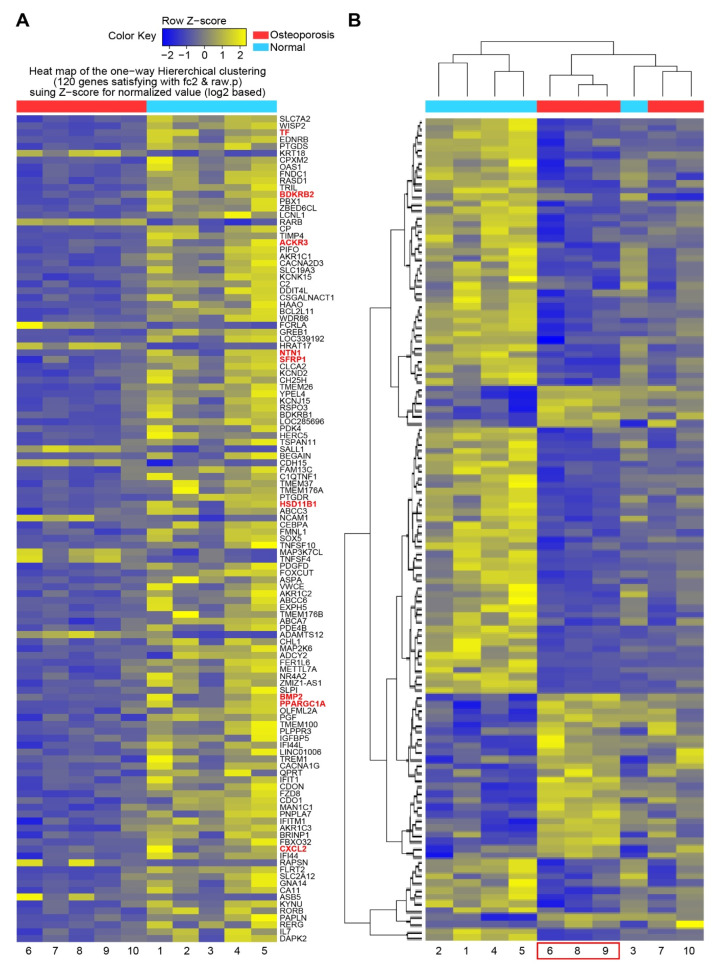
Heatmap of DEG of mesenchymal stromal cells from control and osteoporosis groups. (**A**) Heatmap of one-way hierarchical clustering of all samples showing 120 differentially expressed genes complying with |FC| ≥ 2, independent t-test raw *p*-value < 0.05, and Z-score for normalized value (log2 based). (**B**) Heatmap grouped by similarity of gene expression pattern via hierarchical clustering analysis of significant genes (distance measure = Euclidean distance; linkage method = completeness).

To determine the essential systemic genes related to osteoporosis, DEG analysis was performed on MSCs isolated from each participant. A total of 120 DEGs were identified, and a heatmap of one-way hierarchical clustering was constructed (Figure 1A). Subsequently, 120 DEGs were regrouped by similarity of expression pattern via hierarchical clustering analysis of significant genes. According to the heatmap, three participants in the osteoporosis group (no. 6, 8, and 9) demonstrated similar gene expression patterns, which were similar to that displayed by participant no. 3 in the control group (Figure 1B). This result suggested that the differentiating genes between control subject no. 3 and those commonly upregulated and downregulated (i.e., similar expression patterns) among the other four participants in the control group could be used as genetic factors to classify participant no. 3 as the control subject in further analysis.

### 3.3. RNA Analysis of Bone Remodeling Epigenome

**Figure 2:**
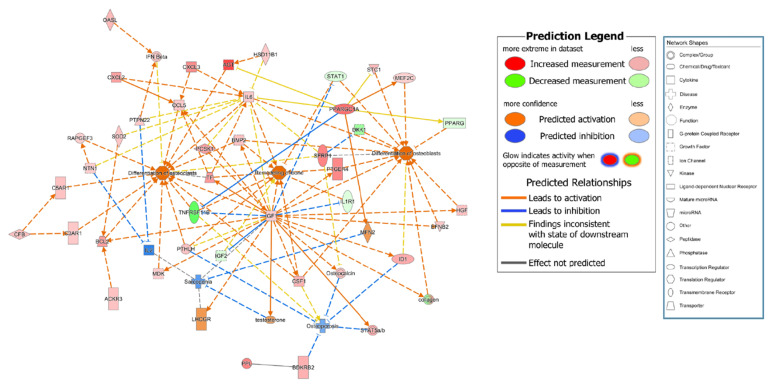
Functional relationship network of genes involved in osteoporosis pathogenesis. Functional relationship network for control subject no. 3 compared with three participants in the osteoporosis group (no. 6, 8, and 9).

To compare the gene expression pattern observed for control subject no. 3 with that of the other participants in the control (excluding the chemical molecules registered in IPA), nine genes related to bone remodeling were selected among the top 120 genes (marked in red in Figure 1A). To connect these nine genes with the bone remodeling epigenome, intermediate molecules expressed in the MSCs of all participants in the control group were added to produce 38 genes. The expression levels of these 38 genes for the participants in the control group are listed in Table 2 and Table S1. The expression of some genes was upregulated for control subject no. 3 compared to that of the other control group participants, while that of other genes was downregulated (Table S2). Specifically, the expressions of BCL2 apoptosis regulator (*BCL2*), bradykinin receptor B2 (*BDKRB2*), insulin-like growth factor 1 (*IGF1*), *IL6*, PPARG coactivator 1 α (*PPARGC1A*), transferrin (*TF*), and 19 other genes were upregulated for control subject no. 3 compared to that of other control group participants, while the expressions of insulin-like growth factor 2 (*IGF2*), interleukin 1 receptor type 1 (*IL1R1*), and *PPARG* were downregulated. In contrast, the expression of myocyte enhancer factor 2C (*MEF2C*) was upregulated for control subject no. 3 but downregulated for the other participants in the control group (Table 3). Direct and indirect relationship networks of the 38 genes related to osteoblasts, osteoclasts, bone remodeling, osteoporosis, and sarcopenia were subsequently generated (Figure 2 and Figure S1). Based on these data, we identified 25 commonly upregulated genes that might be essential systemic genes involved in normal bone remodeling and osteoporosis prevention.

### 3.4. Genetic Relationship among Osteoporosis, Obesity and Diabetes

**Figure 3:**
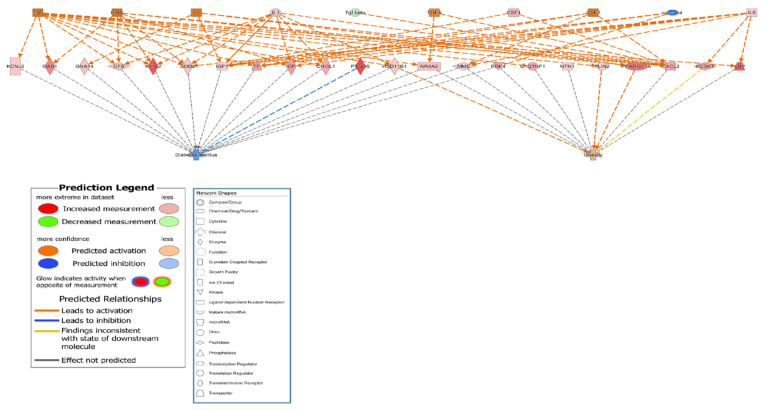
Functional relationship networks of osteoporosis-related genes involved in the canonical pathways of obesity and diabetes development. Functional relationship networks for control subject no. 3 compared with three participants in the osteoporosis group (no. 6, 8, and 9).

To clarify the related pathways among osteoporosis, obesity, and diabetes, the DEGs in obesity-related and diabetes-related networks were identified among the MSCs of all participants in the control group genes using IPA software. A total of 24 osteoporosis-related genes, including *IGF1*, *IL6*, pyruvate dehydrogenase kinase 4 (*PDK4*), *PPARGC1A*, and *TF*, were also associated with obesity and diabetes. All 24 genes were highly expressed with direct or indirect relationships established via intermediate molecules such as calcitriol, colony stimulating factor 2 (*CSF-2*), interferon-γ (*IFN-γ*), *IL1*, *IL4*, transforming growth factor-α (*TGF-α*), transforming growth factor-β (*TGF-β*), and tumor necrosis factor (*TNF*) (Table 4 and Table S3). The relationship networks are illustrated in Figure 3 and Figure S2, and they demonstrate that 24 candidate genes reduced diabetes and increased obesity. These results suggested that the 24 candidate genes might be involved in the development of osteoporosis as well as obesity and diabetes.

### 3.5. Common Related Genes in Osteoporosis, Sarcopenia, Diabetes, and Obesity

**Figure 4:**
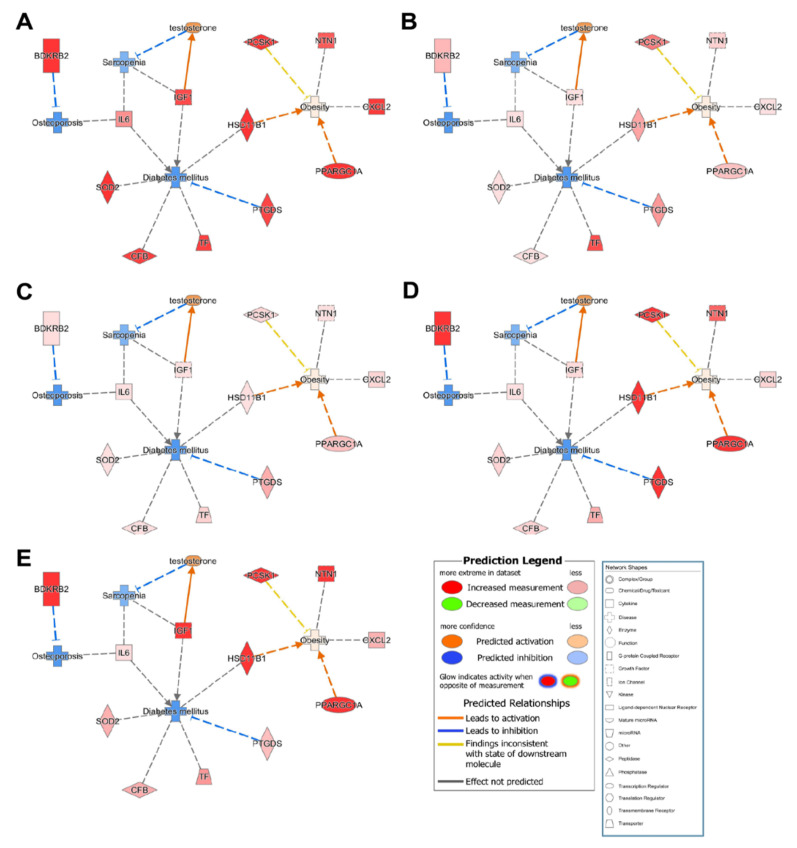
Functional relationship networks shared among osteoporosis-, sarcopenia-, obesity-, and diabetes-related genes. Control group participants no. (**A**) 1, (**B**) 2, (**C**) 3, (**D**) 4, and (**E**) 5.

The IPA results revealed 10 common candidate genes (*CFB, CXCL2, HSD11B1, IGF1, IL6, NTN1, PCSK1, PPARGC1A, SOD2,* and *TF*) that were inhibited in osteoporosis, sarcopenia, and diabetes but activated in obesity for all participants in the control group (Figure 4). Furthermore, IPA data suggested that activation of *HSD11B1* and *PPARGA1A* resulted in obesity, but the other eight candidate genes were not significantly associated with obesity.

## 4. Discussion

MSCs are multipotent cells with the potential to differentiate into multiple lineages, including osteoblasts, adipocytes, and chondroblasts [24,25]. Therefore, MSCs were employed in the present study to investigate the relationship between bone and fat cells. RNA-seq was performed to identify DEGs in MSCs collected from healthy individuals and subjects with osteoporosis [26,27,28,29]. Previous studies have pooled numerous samples together to obtain DEG data during investigations of the overall trends of disease [14,30,31]. In the traditional pooling system, individual specific genes may not be selected or lost by other control samples. In the current study, each of the five samples from the control and osteoporosis groups was analyzed separately to determine the minimum individual character gene groups involved in osteoporosis.

Using IPA software, we identified 25 important systemic genes related to bone remodeling and 24 candidate osteoporosis-related genes associated with obesity and diabetes. Moreover, 10 common genes that influence metabolic disorders such as osteoporosis, sarcopenia, diabetes, and obesity were identified. Most of the candidate genes were related to bone remodeling and/or sarcopenia, diabetes, and obesity, as supported by the results of previous studies. Complement factor B (*CFB*) plays a significant role in the pathogenesis of diabetic retinopathy [32], and there might be a relationship between *CFB* and diabetes onset. Furthermore, *CFB* plays an important role in the differentiation of 3T3-L1 preadipocytes in CFB transgenic mice [33], and *CFB* and *IL6* are associated with the rapid metabolism of glucagon in metabolically compromised subjects [34]. Additionally, *IL6* individually controls osteoblast differentiation and induces bone resorption [35]. Since hypertrophied white adipose tissue produces chemokines, *CXCL2* expression is increased in osteoclastogenesis [36] compared with osteoblastogenesis [37], and white adipose tissue is activated by *CXCL2* to prevent the onset of obesity [38]. *HSD11B1* might be related to BMD [39] and is associated with the onset of type 2 diabetes [40,41]. *IGF-1* is known to enhance muscle mass and alleviate sarcopenia [42], and low serum IGF-1 levels could increase the risk of idiopathic osteoporosis [43,44]. Low IGF-1 levels not only affect BMD but also results in the onset of type 1 or 2 diabetes mellitus [45,46]. *NTN1* is an essential factor involved in the differentiation of osteoclasts [47] and is also involved in osteoblast differentiation [48]. Low *PCSK1* expression might be related to obesity and diabetes [49], and *PPARGC1A* expression can increase obesity risk [50]. *SOD2* increases osteoblast differentiation and bone formation [51,52] and protects neurons in diabetic neuropathy [53]. The expression of *SOD2* is higher in patients with obesity than in healthy individuals [54]. *TF* can repress alkaline phosphatase activities in osteoblasts due to high oxidative stress [55], and increased *TF* expression increases the risk of type 1 and/or type 2 diabetes [56]. Taken together, these findings support that the 10 candidate genes identified in the current study are associated with metabolic disorders.

Osteoporosis commonly occurs with other endocrine system-related diseases, such as obesity and diabetes. Bone and fat cells are known to have a common origin [57], and osteoporosis and diabetes can both be induced by vitamin D deficiency [58]. Moreover, insulin signaling pathway-associated proteins can induce both osteoporosis and diabetes [59]. However, studies that include osteoporosis, sarcopenia, obesity, and diabetes are extremely rare.

In the current study, we analyzed individual differences in MSC gene expression among participants in the control and osteoporosis groups to overcome the disadvantage of previous studies that investigated overall expression trends. Another merit of the present study is that using MSCs to identify osteoporosis-related DEGs enabled us to evaluate the genetic relationship among osteoporosis, sarcopenia, obesity, and diabetes using cells from which osteoblasts and adipocytes commonly originate. Nevertheless, this study has some limitations. First, the mean age difference between the control and osteoporosis groups is statistically significant. The subjects in the control group underwent surgery due to unexpected accidents; therefore, no medical history was available and the statistical difference was unavoidable. Second, we analyzed transcriptional differences associated with osteoporosis-related genes (mRNA). The process of mRNA translation is not always successful; thus, a mechanistic study of these genes is needed. The molecular mechanisms of the identified genes will be explored in future studies.

In summary, we identified the top 25 essential systemic genes involved in osteoporosis using IPA software and the Molecule Activity Predictor tool. Furthermore, we constructed a network of the top 24 genes also associated with diabetes and obesity to verify their association with osteoporosis. The study findings provide insight into the relationship among osteoporosis, obesity, and diabetes, suggesting potential targets for osteoporosis treatment and prevention.

## Figures and Tables

**Table 1:** (**a**) Clinical characteristics of study participants. (**b**) Age-adjusted analysis of bone mineral densities.

**(a)**												
**Subjects**					**Bone Mineral Density**							
	**no.**	**Age (yrs)**	**Height (cm)**	**Weight (kg)**	**Lumbar Spine** **(g/cm^2^)**	**Femur Neck (g/cm^2^)**	**Total Hip (g/cm^2^)**	**Whole BMD (g/cm^2^)**	**ASMI (kg/m^2^)**	**LBM (kg)**	**BMI (kg/m^2^)**	**Diabetes**
Control (*n* = 5)	1	70.0	154.5	75.2	1.023	0.865	0.920	1.068	6.728	37.088	32	No
	2	42.0	164.0	88.1	1.279	1.023	1.102	1.277	7.745	47.568	33	No
	3	64.0	150.1	55.2	1.016	1.022	0.989	0.970	6.133	31.741	25	No
	4	55.0	172.0	70.6	1.269	1.102	1.157	1.380	6.946	47.762	24	No
	5	71.0	150.0	70.3	1.016	0.905	1.134	1.201	6.938	39.237	31	No
Mean		60.4	158.12	71.9	1.121	0.983	1.060	1.179	6.898	40.679	28.77	
Osteoporosis (*n* = 5)	6	85.0	160.0	49.1	0.713	0.519	0.625	0.760	4.339	30.890	19	Yes
	7	71.0	151.0	46.6	0.775	0.461	0.520	0.743	5.472	33.145	20	Yes
	8	79.0	152.0	59.0	0.922	0.558	0.579	-	-	-	26	Yes
	9	80.0	151.0	54.0	0.810	0.636	0.623	0.878	4.613	28.831	24	Yes
	10	72.0	141.0	54.8	0.794	0.696	0.728	0.836	5.384	28.872	28	No
Mean		77.4	151.0	52.7	0.803	0.574	0.615	0.804	4.952	30.435	23.28	
**(b)**												
**Subjects**				**Bone Mineral Density**								
			**no.**	**Lumbar Spine** **(g/cm^2^)**		**Femur Neck** **(g/cm^2^)**		**Total Hip** **(g/cm^2^)**		**Whole BMD** **(g/cm^2^)**		
Control (*n* = 5)			1	1.024		0.866		0.921		1.073		
			2	1.139		0.909		0.978		1.131		
			3	1.082		1.076		1.048		1.045		
			4	1.010		0.892		0.928		1.107		
			5	1.084		0.960		1.194		1.278		
Mean				1.068		0.940		1.014		1.127		
Osteoporosis (*n* = 5)			6	0.632		0.453		0.554		0.678		
			7	0.828		0.504		0.567		0.804		
			8	0.960		0.589		0.613				
			9	0.863		0.679		0.670		1.098		
			10	0.995		0.860		0.906		0.841		
Mean				0.856		0.617		0.662		0.855		
*p*-value				0.0141		0.0039		0.0026		0.0194		

ASMI: appendicular skeletal muscle mass index; LBM: lean body mass; BMI: body mass index.

**Table 2:** Expression log2 fold change values of 38 genes related to osteoblasts, osteoclasts, bone remodeling, osteoporosis, and sarcopenia in mesenchymal stromal cells from participants in the control group based on RNA-sequencing data.

Gene Symbol	no. 1	no. 2	no. 3	no. 4	no. 5
*ACKR3*	6.855	1.439	1.303	3.895	15.063
*AGT*	−2.107	1.005	4.004	−1.015	3.397
*BCL2*	−1.472	1.542	2.091	1.187	−1.068
*BDKRB2*	27.36	3.53	1.683	12.459	17.274
*BMP2*	8.682	−1.198	1.278	2.626	6.76
*C3AR1*	1.315	3.404	1.158	2.263	1.045
*C5AR1*	−2.64	1.496	1.305	−1.334	−2.939
*CCL5*	5.637	2.635	1.199	1.268	4.472
*CFB*	9.544	1.4	1.361	2.173	3.967
*CSF1*	4.442	2.414	1.685	2.024	3.503
*CXCL2*	9.746	1.278	2.029	2.115	3.785
*CXCL3*	81.438	1.572	2.468	10.269	20.713
*DKK1*	2.336	−4.204	−2.932	−1.544	1.073
*EFNB2*	1.194	1.937	1.172	2.068	−1.052
*HGF*	1.221	2.207	1.339	1.817	1.562
*HSD11B1*	30.422	4.501	1.147	18.48	29.309
*ID1*	2.283	1.614	1.799	1.486	5.361
*IGF1*	9.016	1.147	1.068	1.793	14.374
*IGF2*	1.476	3.942	−1.069	5.632	4.821
*IL1R1*	3.726	2.233	−1.03	2.565	3.842
*IL6*	5.484	1.272	1.155	1.505	1.657
*MDK*	1.975	1.966	1.07	2.106	3.017
*MEF2C*	−2.282	−1.75	1.051	−2.7	−2.127
*NTN1*	8.152	2.264	1.058	7.593	9.835
*OASL*	7.487	3.377	1.48	2.564	1.796
*PCSK1*	58.887	6.653	2.041	21.927	77.419
*PPARG*	1.233	2.796	−1.014	2.469	2.021
*PPARGC1A*	19.933	3.017	3.13	12.553	32.307
*PPL*	10.936	9.128	2.676	19.03	19.77
*PTGER4*	−1.016	2.245	2.538	3.785	5.318
*PTPN22*	8.385	3.023	1.19	4.239	7.19
*RAPGEF3*	2.288	2.067	1.051	2.22	4.005
*SFRP1*	6.398	6.344	2.657	6.847	6.662
*SOD2*	12.349	1.219	1.229	2.142	3.923
*STAT1*	1.141	−1.865	−1.121	−2.134	−1.723
*STC1*	12.298	1.987	1.49	2.41	5.001
*TF*	9.482	8.842	2.214	4.156	5.078
*TNFRSF11B*	2.63	−2.201	−4.728	−1.36	−1.556

Entrez gene names and *p*-values are listed in Table S1.

**Table 3:** Commonly upregulated or downregulated genes in mesenchymal stromal cells from participants in the control group.

Commonly Upregulated for Control Subject no. 3 Compared to Other Participants in Control Group (25)	Commonly Downregulated for Control Subject no. 3 Compared to Other Participants in Control Group (0)	Downregulated for Control Subject no. 3 and Upregulated for Other Participants in Control Group (3)	Upregulated for Control Subject no. 3 and Downregulated for Other Participants in Control Group (1)
*ACKR3*	-	*IGF2*	*MEF2C*
*BDKRB2*	-	*IL1R1*	-
*C3AR1*	-	*PPARG*	-
*CCL5*	-	-	-
*CFB*	-	-	-
*CSF1*	-	-	-
*CXCL2*	-	-	-
*CXCL3*	-	-	-
*HGF*	-	-	-
*HSD11B1*	-	-	-
*ID1*	-	-	-
*IGF1*	-	-	-
*IL6*	-	-	-
*MDK*	-	-	-
*NTN1*	-	-	-
*OASL*	-	-	-
*PCSK1*	-	-	-
*PPARGC1A*	-	-	-
*PPL*	-	-	-
*PTPN22*	-	-	-
*RAPGEF3*	-	-	-
*SFRP1*	-	-	-
*SOD2*	-	-	-
*STC1*	-	-	-
*TF*	-	-	-

**Table 4:** Expression log2 fold change values of 24 genes related to osteoporosis, obesity, and diabetes in mesenchymal stromal cells from participants in the control group based on RNA-sequencing data.

Gene Symbol	no. 1	no. 2	no. 3	no. 4	no. 5
*C1QTNF1*	16.527	3.107	1.901	3.946	7.916
*CFB*	9.544	1.4	1.361	2.173	3.967
*CHI3L1*	15.654	2.156	1.85	2.939	16.257
*CP*	14.028	15.237	2.171	10.585	40.727
*CSF1*	4.442	2.414	1.685	2.024	3.503
*CXCL2*	9.746	1.278	2.029	2.115	3.785
*GNA14*	8.773	4.354	1.338	7.26	47.7
*HSD11B1*	30.422	4.501	1.147	18.48	29.309
*IGF1*	9.016	1.147	1.068	1.793	14.374
*IL6*	5.484	1.272	1.155	1.505	1.657
*KCNJ2*	13.25	8.057	1.156	6.693	4.853
*KYNU*	21.468	5.505	3.764	17.825	35.813
*LBP*	47.991	98.045	3.718	66.303	84.066
*MME*	15.395	3.207	1.072	6.601	3.175
*NR4A2*	16.509	4.202	1.354	6.798	7.023
*NTN1*	8.152	2.264	1.058	7.593	9.835
*OAS1*	8.047	3.939	2.701	3.431	7.107
*PCSK1*	58.887	6.653	2.041	21.927	77.419
*PDK4*	10.246	1.569	2.154	1.917	10.588
*PLIN2*	6.437	1.52	1.065	1.003	1.366
*PPARGC1A*	19.933	3.017	3.13	12.553	32.307
*PTGDS*	9.258	5.15	4.065	13.654	3.19
*SOD2*	12.349	1.219	1.229	2.142	3.923
*TF*	9.482	8.842	2.214	4.156	5.078

Entrez gene names and *p*-values are listed in Table S3.

## Data Availability

All relevant data are included in the manuscript.

## Supplementary Materials

The following are available online at https://www.mdpi.com/article/10.3390/genes13030459/s1, Figure S1: Functional relationship networks of genes associated with the canonical pathways related to osteoporosis development in control group participants; Figure S2: Functional relationship networks of genes associated with the canonical pathways related to obesity and diabetes onset in control group participants; Table S1: RNA-sequencing data of 38 genes related to osteoblasts, osteoclasts, bone remodeling, osteoporosis, and sarcopenia in mesenchymal stromal cells among participants in the control group; Table S2: Genes commonly upregulated or downregulated for control subject no. 3 according to RNA-sequencing data, compared to control participants no. (A) 1, (B) 2, (C) 4, and (D) 5; Table S3: Expression of 24 genes related to obesity and diabetes in mesenchymal stromal cells among participants in the control group based on RNA-sequencing data.
