# Supplementary material for: Gene Network Analysis for Osteoporosis, Sarcopenia, Diabetes, and Obesity in Human Mesenchymal Stromal Cells
Source: Genes (Basel). 2022 Mar 3;13(3):459. doi: 10.3390/genes13030459 (PMC8953569; doi:10.3390/genes13030459)
Supplement: Supplementary file 1 [file genes-13-00459-s001.zip › genes-1556314 -supplementary figures revised.pdf]

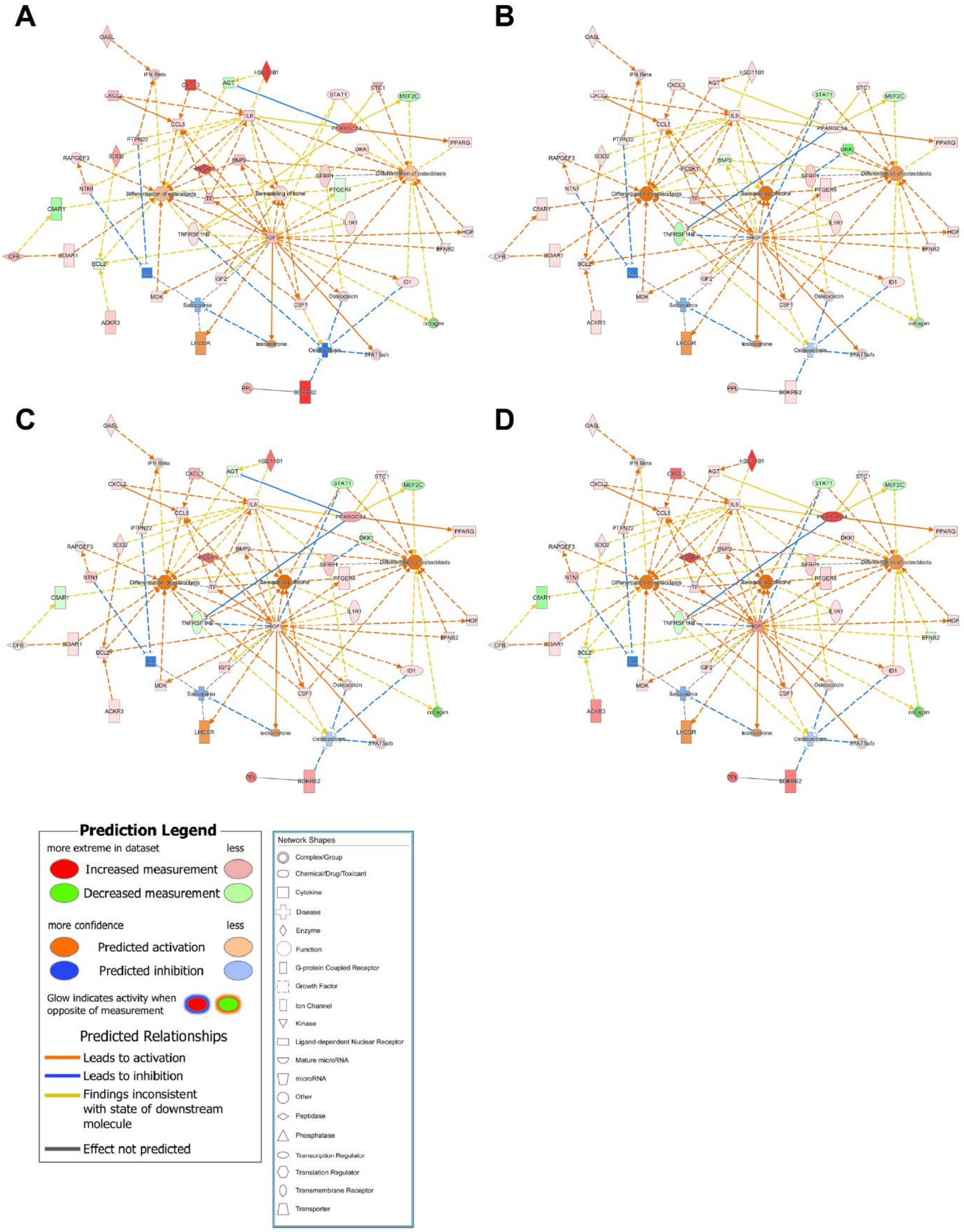

Figure S1. Functional relationship networks of genes associated with the canonical pathways related to osteoporosis development in control participants. Control participants (A) no. 1, (B) no. 2, (C) no. 4, and (D) no. 5.

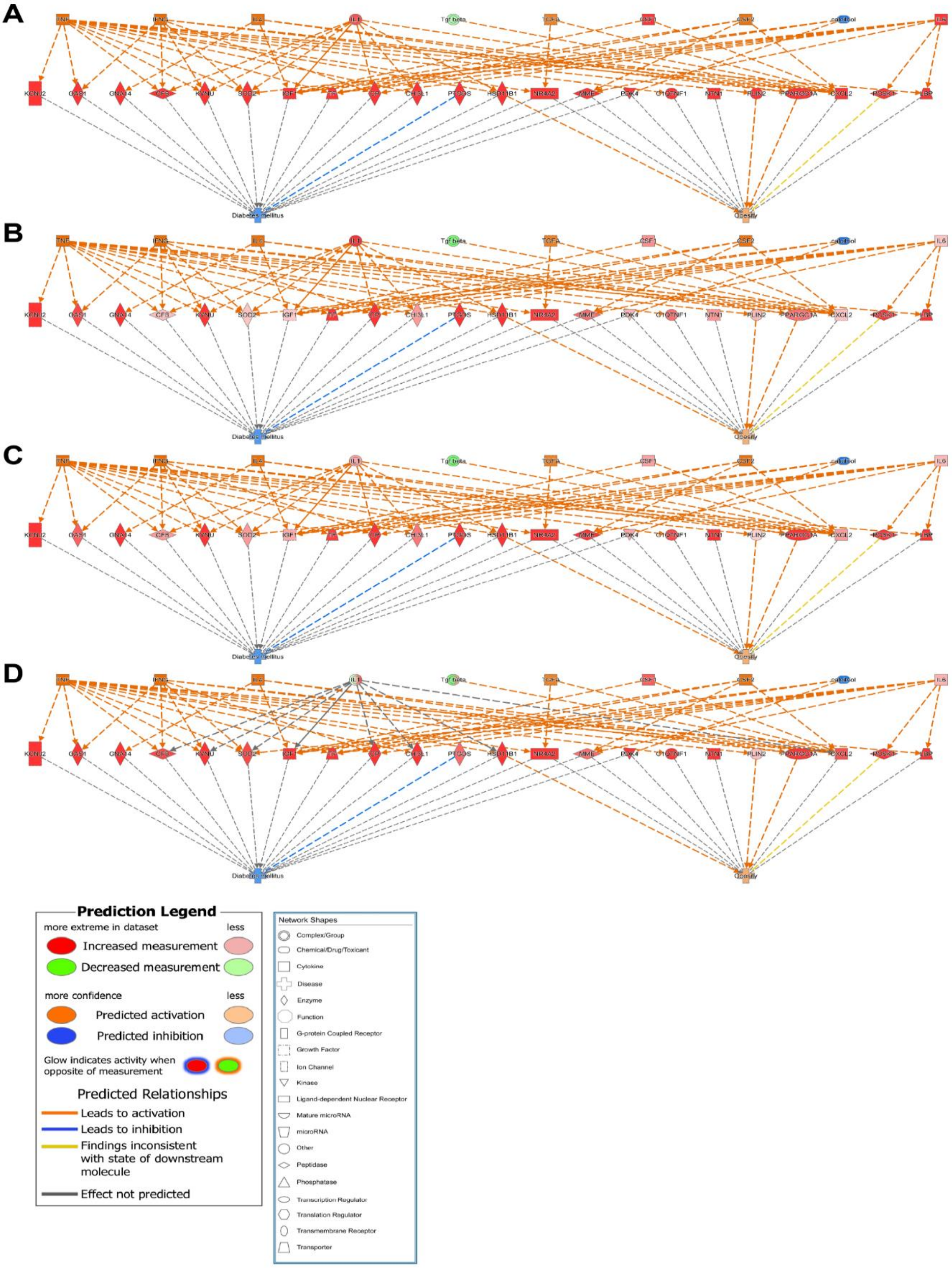

Figure S2. Function relationship networks of genes associated with the canonical pathways related to obesity and diabetes onset in control participants. Control participants no. (A) 1, (B) 2, (C) 4, and (D) 5.
