# Supplementary material for: Gene Network Analysis for Osteoporosis, Sarcopenia, Diabetes, and Obesity in Human Mesenchymal Stromal Cells
Source: Genes (Basel). 2022 Mar 3;13(3):459. doi: 10.3390/genes13030459 (PMC8953569; doi:10.3390/genes13030459)
Supplement: Supplementary file 1 [file genes-13-00459-s001.zip › genes-1556314-Supplementary tables revised.pdf]

Supplementary Tables

Table S1. RNA-sequencing data of 38 genes related to osteoblasts, osteoclasts, bone remodeling, osteoporosis, and sarcopenia in mesenchymal stromal cells among participants in the control group.

		no .1		no .2		no .3		no .4		no .5	
**Gene symbol**	**Entrez Gene Name**	**Expr Log Ratio**	**Expr *p*-value**	**Expr Log Ratio**	**Expr *p*-value**	**Expr Log Ratio**	**Expr *p*-value**	**Expr Log Ratio**	**Expr *p*-value**	**Expr Log Ratio**	**Expr *p*-value**
ACKR3	atypical chemokine receptor 3	6.86	9.29×10^{-12}	1.44	1.99×10^{-1}	1.30	3.50×10^{-1}	3.90	1.51×10^{-6}	15.06	1.14×10^{-19}
AGT	angiotensinogen	−2.11	1.12×10^{-2}	1.01	9.85×10^{-1}	4.00	2.13×10^{-6}	−1.02	9.58×10^{-1}	3.40	2.00×10^{-4}
BCL2	BCL2 apoptosis regulator	−1.47	1.66×10^{-1}	1.54	1.16×10^{-1}	2.09	7.37×10^{-3}	1.19	5.36×10^{-1}	−1.07	8.24×10^{-1}
BDKRB2	bradykinin receptor B2	27.36	1.08×10^{-52}	3.53	6.82×10^{-9}	1.68	1.76×10^{-2}	12.46	2.82×10^{-31}	17.27	2.08×10^{-34}
BMP2	bone morphogenetic protein 2	8.68	3.29×10^{-13}	−1.20	5.51×10^{-1}	1.28	4.17×10^{-1}	2.63	1.26×10^{-3}	6.76	4.85×10^{-10}
C3AR1	complement C3a receptor 1	1.32	4.26×10^{-1}	3.40	4.33×10^{-4}	1.16	6.70×10^{-1}	2.26	1.85×10^{-2}	1.05	9.15×10^{-1}
C5AR1	complement C5a receptor 1	−2.64	1.13×10^{-3}	1.50	1.64×10^{-1}	1.31	3.58×10^{-1}	−1.33	3.25×10^{-1}	−2.94	6.29×10^{-4}
CCL5	C-C motif chemokine ligand 5	5.64	1.10×10^{-6}	2.64	6.31×10^{-3}	1.20	6.08×10^{-1}	1.27	5.03×10^{-1}	4.47	4.87×10^{-4}
CFB	complement factor B	9.54	9.42×10^{-52}	1.40	2.59×10^{-2}	1.36	4.20×10^{-2}	2.17	2.61×10^{-7}	3.97	1.12×10^{-21}
CSF1	colony stimulating factor 1	4.44	3.40×10^{-17}	2.41	6.33×10^{-7}	1.69	3.20×10^{-3}	2.02	6.8×10^{-5}	3.50	1.00×10^{-11}
CXCL2	C-X-C motif chemokine ligand 2	9.75	1.09×10^{-10}	1.28	4.87×10^{-1}	2.03	4.48×10^{-2}	2.12	3.37×10^{-2}	3.79	2.17×10^{-3}
CXCL3	C-X-C motif chemokine ligand 3	81.44	7.09×10^{-51}	1.57	1.35×10^{-1}	2.47	2.62×10^{-3}	10.27	3.02×10^{-15}	20.71	4.91×10^{-17}
DKK1	dickkopf WNT signaling pathway inhibitor 1	2.34	1.72×10^{-3}	−4.20	1.23×10^{-7}	−2.93	7.36×10^{-5}	−1.54	1.09×10^{-1}	1.07	8.16×10^{-1}
EFNB2	ephrin B2	1.19	5.32×10^{-1}	1.94	1.97×10^{-2}	1.17	5.75×10^{-1}	2.07	1.04×10^{-2}	−1.05	8.73×10^{-1}

HGF	hepatocyte growth factor	1.22	5.68 x 10^{-1}	2.21	2.40 x 10^{-2}	1.34	4.05 x 10^{-1}	1.82	8.84 x 10^{-2}	1.56	2.05 x 10^{-1}
HSD11B1	hydroxysteroid 11-beta dehydrogenase 1	30.42	1.71 x 10^{-30}	4.50	5.62 x 10^{-7}	1.15	6.60 x 10^{-1}	18.48	1.29 x 10^{-22}	29.31	8.69 x 10^{-26}
ID1	inhibitor of DNA binding 1, HLH protein	2.28	2.79 x 10^{-3}	1.61	8.32 x 10^{-2}	1.80	3.35 x 10^{-2}	1.49	1.52 x 10^{-1}	5.36	2.84 x 10^{-9}
IGF1	insulin like growth factor 1	9.02	2.86 x 10^{-10}	1.15	6.94 x 10^{-1}	1.07	8.51 x 10^{-1}	1.79	9.45 x 10^{-2}	14.37	3.56 x 10^{-12}
IGF2	insulin like growth factor 2	1.48	2.20 x 10^{-1}	3.94	1.50 x 10^{-5}	−1.07	8.34 x 10^{-1}	5.63	4.87 x 10^{-8}	4.82	1.72 x 10^{-5}
IL6	interleukin 6	5.48	1.44 x 10^{-6}	1.27	4.95 x 10^{-1}	1.16	6.83 x 10^{-1}	1.51	2.47 x 10^{-1}	1.66	2.44 x 10^{-1}
IL1R1	interleukin 1 receptor type 1	3.73	2.24 x 10^{-11}	2.23	4.44 x 10^{-5}	−1.03	8.82 x 10^{-1}	2.57	1.68 x 10^{-6}	3.84	1.08 x 10^{-10}
MDK	midkine	1.98	3.45 x 10^{-2}	1.97	3.57 x 10^{-2}	1.07	8.34 x 10^{-1}	2.11	2.07 x 10^{-2}	3.02	3.02 x 10^{-3}
MEF2C	myocyte enhancer factor 2C	−2.28	1.44 x 10^{-3}	−1.75	2.96 x 10^{-2}	1.05	8.47 x 10^{-1}	−2.70	1.29 x 10^{-4}	−2.13	4.67 x 10^{-3}
NTN1	netrin 1	8.15	2.19 x 10^{-11}	2.26	9.80 x 10^{-3}	1.06	8.61 x 10^{-1}	7.59	1.02 x 10^{-10}	9.84	2.20 x 10^{-12}
OASL	2'-5'-oligoadenylate synthetase like	7.49	2.63 x 10^{-9}	3.38	3.28 x 10^{-4}	1.48	2.50 x 10^{-1}	2.56	5.6 x 10^{-3}	1.80	1.68 x 10^{-1}
PCSK1	proprotein convertase subtilisin/kexin type 1	58.89	6.25 x 10^{-39}	6.65	1.76 x 10^{-9}	2.04	2.65 x 10^{-2}	21.93	6.03 x 10^{-23}	77.42	3.62 x 10^{-39}
PPARG	peroxisome proliferator activated receptor gamma	1.23	4.90 x 10^{-1}	2.80	6.42 x 10^{-4}	-1.01	9.63 x 10^{-1}	2.47	2.72 x 10^{-3}	2.02	3.67 x 10^{-2}
PPARGC1A	PPARG coactivator 1 alpha	19.93	1.06 x 10^{-18}	3.02	1.23 x 10^{-3}	3.13	8.48 x 10^{-4}	12.55	9.02 x 10^{-14}	32.31	1.25 x 10^{-21}
PPL	periplakin	10.94	8.19 x 10^{-14}	9.13	5.06 x 10^{-12}	2.68	2.17 x 10^{-3}	19.03	3.57 x 10^{-20}	19.77	1.18 x 10^{-15}
PTGER4	prostaglandin E receptor 4	−1.02	9.39 x 10^{-1}	2.25	1.11 x 10^{-4}	2.54	8.54 x 10^{-6}	3.79	1.93 x 10^{-10}	5.32	2.16 x 10^{-15}
PTPN22	protein tyrosine phosphatase non-receptor type 22	8.39	9.75 x 10^{-10}	3.02	1.49 x 10^{-3}	1.19	6.18 x 10^{-1}	4.24	3.36 x 10^{-5}	7.19	9.98 x 10^{-7}
RAPGEF3	Rap guanine nucleotide exchange factor 3	2.29	9.04 x 10^{-3}	2.07	2.19 x 10^{-2}	1.05	8.76 x 10^{-1}	2.22	1.19 x 10^{-2}	4.01	1.77 x 10^{-5}
SFRP1	secreted frizzled related protein 1	6.40	1.01 x 10^{-8}	6.34	1.16 x 10^{-8}	2.66	2.57 x 10^{-3}	6.85	2.85 x 10^{-9}	6.66	1.30 x 10^{-5}

SOD2	superoxide dismutase 2	12.35	4.37×10^{-71}	1.22	1.63×10^{-1}	1.23	1.47×10^{-1}	2.14	7.49×10^{-8}	3.92	9.87×10^{-23}
STAT1	signal transducer and activator of transcription 1	1.14	5.12×10^{-1}	−1.87	1.95×10^{-3}	-1.12	5.70×10^{-1}	−2.13	1.66×10^{-4}	−1.72	1.13×10^{-2}
STC1	stanniocalcin 1	12.30	9.38×10^{-13}	1.99	5.08×10^{-2}	1.49	2.57×10^{-1}	2.41	1.23×10^{-2}	5.00	5.46×10^{-6}
TF	transferrin	9.48	1.06×10^{-11}	8.84	4.41×10^{-11}	2.21	1.68×10^{-2}	4.16	1.74×10^{-5}	5.08	6.04×10^{-5}
TNFRSF11B	TNF receptor superfamily member 11b	2.63	4.68×10^{-9}	−2.20	1.88×10^{-6}	−4.73	1.02×10^{-20}	−1.36	6.29×10^{-2}	−1.56	9.13×10^{-3}

Entrez gene name, expression levels, and *p*-values of genes are listed for each sample. All data leave 2 digits after the decimal point.

Table S2. Genes commonly upregulated or downregulated in control subject no. 3 according to RNA-sequencing data, compared to control participants no. 1 (A), 2 (B), 4 (C), and 5 (D).

A

Common up (27)	Common down (0)	no.3 down & no.1 up (6)	no.3 up & no.1 down (5)
ACKR3	—	*DKK1*	*AGT*
BDKRB2		*IGF2*	*BCL2*
BMP2		*IL1R1*	*C5AR1*
C3AR1		*PPARG*	*MEF2C*
CCL5		*STAT1*	*PTGER4*
CFB		*TNFRSF11B*	
CSF1			
CXCL2			
CXCL3			
EFNB2			
HGF			
HSD11B1			
ID1			
IGF1			
IL6			
MDK			
NTN1			
OASL			
PCSK1			
PPARGC1A			
PPL			
PTPN22			
RAPGEF3			

B

Common up (30)	Common down (3)	no.3 down & no.2 up (3)	no.3 up & no.2 down (2)
ACKR3	*DKK1*	*IGF2*	*BMP2*
AGT	*STAT1*	*IL1R1*	*MEF2C*
BCL2	*TNFRSF11B*	*PPARG*	
BDKRB2			
C3AR1			
C5AR1			
CCL5			
CFB			
CSF1			
CXCL2			
CXCL3			
EFNB2			
HGF			
HSD11B1			
ID1			
IGF1			
IL6			
MDK			
NTN1			
OASL			
PCSK1			
PPARGC1A			
PPL			

SFRP1			
SOD2			
STC1			
TF			

PTGER4			
PTPN22			
RAPGEF3			
SFRP1			
SOD2			
STC1			
TF	—	—	—

C

Common up (29)	Common down (3)	no.3 down & no.4 up (3)	no.3 up & no.4 down (3)
ACKR3	*DKK1*	*IGF2*	*AGT*
BCL2	*STAT1*	*IL1R1*	*C5AR1*
BDKRB2	*TNFRSF11B*	*PPARG*	*MEF2C*
BMP2			
C3AR1			
CCL5			
CFB			
CSF1			
CXCL2			
CXCL3			
EFNB2			
HGF			
HSD11B1			
ID1			
IGF1			
IL6			
MDK			

D

Common up (28)	Common down (2)	no.3 down & no.5 up (4)	no.3 up & no.5 down (4)
ACKR3	*STAT1*	*DKK1*	*BCL2*
AGT	*TNFRSF11B*	*IGF2*	*C5AR1*
BDKRB2		*IL1R1*	*EFNB2*
BMP2		*PPARG*	*MEF2C*
C3AR1			
CCL5			
CFB			
CSF1			
CXCL2			
CXCL3			
HGF			
HSD11B1			
ID1			
IGF1			
IL6			
MDK			
NTN1			

NTN1
OASL
PCSK1
PPARGC1A
PPL
PTGER4
PTPN22
RAPGEF3
SFRP1
SOD2
STC1
TF

OASL
PCSK1
PPARGC1A
PPL
PTGER4
PTPN22
RAPGEF3
SFRP1
SOD2
STC1
TF

Table S3. Expression of 24 genes related to obesity and diabetes in mesenchymal stromal cells among participants in the control group based on RNA-sequencing data.

		no .1		no .2		no .3		no .4		no .5	
**Gene Symbol**	**Entrez Gene Name**	**Expr Log Ratio**	**Expr *p*-value**	**Expr Log Ratio**	**Expr *p*-value**	**Expr Log Ratio**	**Expr *p*-value**	**Expr Log Ratio**	**Expr *p*-value**	**Expr Log Ratio**	**Expr *p*-value**
C1QTNF1	C1q and TNF related 1	16.53	2.45×10^{-63}	3.11	1.55×10^{-11}	1.90	1.50×10^{-4}	3.95	3.33×10^{-16}	7.92	8.22×10^{-35}
CFB	complement factor B	9.54	9.42×10^{-52}	1.40	2.59×10^{-2}	1.36	4.20×10^{-2}	2.17	2.61×10^{-7}	3.97	1.12×10^{-21}
CHI3L1	chitinase 3 like 1	15.65	3.20×10^{-15}	2.16	2.77×10^{-2}	1.85	7.79×10^{-2}	2.94	2.00×10^{-3}	16.26	1.59×10^{-10}
CP	ceruloplasmin	14.03	1.96×10^{-14}	15.24	2.90×10^{-15}	2.17	2.53×10^{-2}	10.59	8.23×10^{-12}	40.73	6.12×10^{-22}
CSF1	colony stimulating factor 1	4.44	3.40×10^{-17}	2.41	6.33×10^{-7}	1.69	3.20×10^{-3}	2.02	6.80×10^{-5}	3.50	1.00×10^{-11}
CXCL2	C-X-C motif chemokine ligand 2	9.75	1.09×10^{-10}	1.28	4.87×10^{-1}	2.03	4.48×10^{-2}	2.12	3.37×10^{-2}	3.79	2.17×10^{-3}
GNA14	G protein subunit alpha 14	8.77	4.62×10^{-11}	4.35	8.63×10^{-6}	1.34	3.86×10^{-1}	7.26	1.92×10^{-9}	47.70	2.29×10^{-43}
HSD11B1	hydroxysteroid 11-beta dehydrogenase 1	30.42	1.71×10^{-30}	4.50	5.62×10^{-7}	1.15	6.60×10^{-1}	18.48	1.29×10^{-22}	29.31	8.69×10^{-26}
IGF1	insulin like growth factor 1	9.02	2.86×10^{-10}	1.15	6.94×10^{-1}	1.07	8.51×10^{-1}	1.79	9.45×10^{-2}	14.37	3.56×10^{-12}
IL6	interleukin 6	5.48	1.44×10^{-6}	1.27	4.95×10^{-1}	1.16	6.83×10^{-1}	1.51	2.47×10^{-1}	1.66	2.44×10^{-1}
KCNJ2	potassium inwardly rectifying channel subfamily J member 2	13.25	1.87×10^{-16}	8.06	3.16×10^{-11}	1.16	6.56×10^{-1}	6.69	1.66×10^{-9}	4.85	5.07×10^{-5}
KYNU	kynureninase	21.47	1.94×10^{-20}	5.51	2.70×10^{-7}	3.76	6.62×10^{-5}	17.83	3.25×10^{-18}	35.81	1.14×10^{-21}
LBP	lipopolysaccharide binding protein	47.99	2.49×10^{-41}	98.05	1.03×10^{-57}	3.72	1.34×10^{-5}	66.30	2.21×10^{-48}	84.07	2.62×10^{-39}
MME	membrane metalloendopeptidase	15.40	3.13×10^{-48}	3.21	6.31×10^{-10}	1.07	7.19×10^{-1}	6.60	9.86×10^{-24}	3.18	4.14×10^{-8}
NR4A2	nuclear receptor subfamily 4 group A member 2	16.51	1.72×10^{-40}	4.20	1.42×10^{-11}	1.35	1.68×10^{-1}	6.80	1.52×10^{-19}	7.02	5.13×10^{-18}
NTN1	netrin 1	8.15	2.19×10^{-11}	2.26	9.80×10^{-3}	1.06	8.61×10^{-1}	7.59	1.02×10^{-10}	9.84	2.20×10^{-12}
OAS1	2'-5'-oligoadenylate synthetase 1	8.05	1.79×10^{-9}	3.94	7.73×10^{-5}	2.70	4.21×10^{-3}	3.43	3.82×10^{-4}	7.11	1.57×10^{-6}
PCSK1	proprotein convertase subtilisin/kexin type 1	58.89	6.25×10^{-39}	6.65	1.76×10^{-9}	2.04	2.65×10^{-2}	21.93	6.03×10^{-23}	77.42	3.62×10^{-39}
PDK4	pyruvate dehydrogenase kinase 4	10.25	8.95×10^{-13}	1.57	1.69×10^{-1}	2.15	1.91×10^{-2}	1.92	4.71×10^{-2}	10.59	2.36×10^{-11}
PLIN2	perilipin 2	6.44	3.04×10^{-36}	1.52	4.82×10^{-3}	1.07	6.74×10^{-1}	1.00	9.85×10^{-1}	1.37	3.76×10^{-2}
PPARGC1A	PPARG coactivator 1 alpha	19.93	1.06×10^{-18}	3.02	1.23×10^{-3}	3.13	8.48×10^{-4}	12.55	9.02×10^{-14}	32.31	1.25×10^{-21}

PTGDS	prostaglandin D2 synthase	9.26	1.78×10^{-25}	5.15	1.61×10^{-14}	4.07	5.27×10^{-11}	13.65	1.48×10^{-34}	3.19	1.91×10^{-6}
SOD2	superoxide dismutase 2	12.35	4.37×10^{-71}	1.22	1.63×10^{-1}	1.23	1.47×10^{-1}	2.14	7.49×10^{-8}	3.92	9.87×10^{-23}
TF	transferrin	9.48	1.06×10^{-11}	8.84	4.41×10^{-11}	2.21	1.68×10^{-2}	4.16	1.74×10^{-5}	5.08	6.04×10^{-5}

Entrez gene name, expression levels, and *p*-values of genes are listed for each sample. All data leave 2 digits after the decimal point.
